# Efficacy of Lamivudine Plus Dolutegravir vs Dolutegravir-Based 3-Drug Regimens in People With HIV Who Are Virologically Suppressed

**DOI:** 10.1093/ofid/ofae198

**Published:** 2024-04-10

**Authors:** Alberto Borghetti, Arturo Ciccullo, Francesca Lombardi, Diana Giannarelli, Rosa Anna Passerotto, Francesco Lamanna, Antonella Carcagnì, Damiano Farinacci, Alex Dusina, Gianmaria Baldin, Maurizio Zazzi, Simona Di Giambenedetto

**Affiliations:** Fondazione Policlinico Universitario A. Gemelli IRCCS, UOC Malattie Infettive, Roma, Italia; Unit of Infectious Diseases, San Salvatore Hospital, L’Aquila, Italy; Dipartimento di Sicurezza e Bioetica Sezione Malattie Infettive, Università Cattolica del Sacro Cuore, Roma, Italia; Facility of Epidemiology and Biostatistics–Gemelli Generator, Fondazione Policlinico Universitario A. Gemelli IRCCS, Rome, Italy; Dipartimento di Sicurezza e Bioetica Sezione Malattie Infettive, Università Cattolica del Sacro Cuore, Roma, Italia; Dipartimento di Sicurezza e Bioetica Sezione Malattie Infettive, Università Cattolica del Sacro Cuore, Roma, Italia; Facility of Epidemiology and Biostatistics–Gemelli Generator, Fondazione Policlinico Universitario A. Gemelli IRCCS, Rome, Italy; Fondazione Policlinico Universitario A. Gemelli IRCCS, UOC Malattie Infettive, Roma, Italia; Fondazione Policlinico Universitario A. Gemelli IRCCS, UOC Malattie Infettive, Roma, Italia; Fondazione Policlinico Universitario A. Gemelli IRCCS, UOC Malattie Infettive, Roma, Italia; Department of Medical Biotechnologies, University of Siena, Siena, Italy; Fondazione Policlinico Universitario A. Gemelli IRCCS, UOC Malattie Infettive, Roma, Italia; Dipartimento di Sicurezza e Bioetica Sezione Malattie Infettive, Università Cattolica del Sacro Cuore, Roma, Italia

**Keywords:** HIV, lamivudine/dolutegravir, three-drug regimen, target trial emulation, virologically suppressed

## Abstract

**Background:**

Lamivudine + dolutegravir maintenance dual therapy (DT) could be less effective than 3-drug therapy (TT) in the context of resistance-associated mutations to nucleoside reverse transcriptase inhibitors (NRTIs). The ARCA database was queried to test this hypothesis with a trial emulation strategy.

**Methods:**

People with HIV taking 2 NRTIs plus a protease inhibitor or a non-NRTI who switched to DT or dolutegravir-based TT were followed up from the first HIV RNA <50 copies/mL (baseline) to virologic failure (VF; ie, 2 consecutive HIV RNA ≥50 copies/mL or 1 HIV RNA ≥200 copies/mL). Those switching to DT within 6 months were assigned to the treatment arm and all other patients to the control arm. Each participant was also cloned, assigned to the opposite strategy, and censored at the time of deviation from that strategy. Using inverse probability of censoring weight Cox regression models, we calculated hazard ratios of VF for DT vs TT stratified for the presence of resistance-associated mutations.

**Results:**

Overall 626 people were analyzed: 204 with DT and 422 with TT (73% men; mean age, 44 years). Ten and 31 VFs occurred with DT and TT, respectively, over a median 5.8 years. When compared with a fully active TT, the DT had similar efficacy (adjusted hazard ratio, 0.88; 95% CI, .29–2.61; *P* = .812) when full susceptibility was confirmed at historical genotype. When previous M184V/I was present in both groups, the risk of VF was higher for DT vs TT but was not statistically significant (adjusted hazard ratio, 3.06; 95% CI, .45–20.84; *P* = .252).

**Conclusions:**

DT was not associated with a significantly higher risk of VF than dolutegravir-based TT.

## Teaser

A trial emulation study based on real-world data showed similar virologic efficacy of lamivudine + dolutegravir as compared with 3-drug regimens among people with HIV who were virologically suppressed.

In recent years, dolutegravir-based 2-drug regimens have been approved by all international guidelines for the management of people with HIV [1–3], with lamivudine plus dolutegravir being the first strategy to be recommended as a first-line regimen and switch strategy in the setting of virologic suppression. In the latter context, lamivudine plus dolutegravir has been approved by international guidelines [1–3] due to the robust evidence from randomized trials [4, 5]. Yet, populations enrolled in randomized controlled trials usually differ from those from clinical practice; therefore, real-world studies are needed to generalize trial results to more complex settings, such as those of patients with previous virologic failures (VFs) or with historical genotypic resistance test results showing archived resistance mutations to study drugs. Several observational [6] studies have been conducted to assess the risk of failure in patients switching in the context of archived resistance-associated mutations (RAMs) to lamivudine (particularly M184V/I) or previous failures to nucleoside reverse transcriptase inhibitors (NRTIs), but so far none of them has reached a definite conclusion. In fact, while some large studies [7, 8] and a small pilot trial [9] have not found an increased risk of failure of dual therapy (DT) in the setting of previously detected RAMs, others have highlighted a possible causal role of M184V/I in increasing the rates of VF of the DT, especially when this mutation is associated with thymidine analogue mutations (TAMs) [10] or with a reduced time of virologic suppression before the switch [11, 12]. Another open question is whether a compromised backbone could influence the efficacy of a DT more than a 3-drug regimen (triple therapy [TT]) composed of 2 NRTIs plus dolutegravir. So far, studies comparing DT with TT [13, 14] suggested a reduced efficacy of lamivudine plus dolutegravir as compared with TT when at least low-level resistance to NRTIs was present at historical genotype. However, comparing retrospectively these 2 strategies could be difficult despite adequate adjustment for confounders, since a possible immortal time bias, emerging from the usually longer time of virologic suppression for patients switching to DT, could be present.

The objective of the present study is to retrospectively compare the efficacy of TT based on 2 NRTIs plus dolutegravir and DT based on lamivudine and dolutegravir, after correcting for the immortal time bias as proposed by Hernán [15], within a multicenter Italian cohort of people with HIV: the Antiviral Response Cohort Analysis (ARCA).

## MATERIALS AND METHODS

### Study Design

This historical cohort study was performed within the ARCA database [16], a longitudinal cohort including >40 000 people with HIV followed at different clinical centers in Italy. More precisely, ARCA is a publicly available database founded in 1995, which includes genotypic resistance test results, viroimmunologic data, and therapeutic histories, as well as clinical and demographic factors.

### Population

Patients undergoing TT with 2 NRTIs plus a protease inhibitor or a nonnucleoside reverse transcriptase inhibitor (NNRTI) who switched to dolutegravir plus 2 NRTIs (TT group) or to lamivudine plus dolutegravir (DT group) between December 2014 and June 2020 were eligible for study analysis if they had HIV RNA <50 copies/mL at the time of the switch and at least 1 follow-up HIV RNA determination after the switch. Exclusion criteria were the presence of a positive hepatitis B surface antigen serostatus, previous treatment with dolutegravir, and the absence of at least 1 previous genotypic resistance test result on HIV RNA.

### Objective

The outcome of the study was the time to VF (defined as 2 consecutive HIV RNA ≥50 copies/mL or 1 HIV RNA ≥200 copies/mL) during a DT regimen as compared with a TT.

Relation of the treatment groups (DT or TT) with failure was explored considering the presence or absence of RAMs. Particularly, treatment regimens were stratified according to 2 variables: (1) a dichotomic one defining the presence or absence of at least 1 potential low-level resistance to the NRTIs employed in the regimen, calculated through the Stanford algorithm [17]; (2) a dummy variable, including the absence of RAMs, the presence of TAMs without M184V/I, M184V/I without TAMs, M184V/I with TAMs, and other RAMs to NRTIs. Since a genotypic resistance test for integrase inhibitors was not available at baseline, we assumed full susceptibility to dolutegravir because no previous failures with integrase inhibitors was documented in this cohort.

### Statistical Analysis

At first, a crude comparison between cumulative incidences of VF during a DT or TT was performed by Kaplan-Meier estimator and log-rank test.

However, as it clearly emerged in the article from Hernán [15], a direct comparison of DT and TT cannot be performed straightforwardly with observational data sets, even after performing analysis with multiple adjustments. In fact, only people with a long duration of viral suppression are usually considered for switching to a DT, and this could lead to a sort of immortal time bias, since these patients could represent a more selected population at lower risk of VF. This kind of bias is not present in randomized trials, since patients are classified by the treatment to which they are assigned and not the treatment to which they will be switched. In the hypothetical trial that we wanted to emulate, the randomization would have been to 1 of the 2 arms: *intervention*, a switch to DT within 6 months from virologic suppression; *control*, no switch to DT or a switch after 6 months from virologic suppression. The 6-month grace period (ie, the period during which we considered feasible for a clinician to consider a switch to DT) was an arbitrary choice based on the aim to understand the impact of a short time of virologic suppression before switching to a DT, especially if RAMs are present at historical genotype.

A 3-step procedure was proposed by Hernán [15] to emulate a randomized trial based on observational data: the first step is cloning patients and assigning them to different treatment strategies; the second step is to censor them when they deviate from the assigned treatment strategy; the third step is to perform inverse probability weighting to adjust for the potential selection bias introduced by censoring.

Translating this procedure to our observational data set, we considered that patients starting a TT or a DT are more comparable when reaching virologic suppression during a TT, since from that point they are potentially eligible for optimizing their treatment. We therefore chose the first HIV RNA <50 copies/mL during a TT with a protease inhibitor or NNRTI as the baseline for our observation. Following the indication provided by Maringe et al [18], based on Hernán's considerations, patients were followed from that moment up to VF or to censor (ie, death, switch to another therapy, or the last available HIV RNA).

In the primary analysis, we considered 6 months of viral suppression as the grace period during which a patient could be assigned to a DT. We assigned people switching to DT within the grace period to the treatment arm and people not switching to DT or switching to 2 NRTIs plus dolutegravir to the control arm. Each participant was then cloned—that is, assigned to the opposite strategy and censored at the time of deviation from that strategy. By using a Cox model adjusted for baseline confounders that could have led to a different censoring time (ie, age, gender, ethnicity, cumulative duration of antiviral exposure, nadir CD4 count, zenith HIV RNA, HIV viral subtype, number of previous VFs, resistance mutations at historical genotype, hepatitis C virus serostatus, previous AIDS-defining event), we estimated the inverse probability of censoring weight (IPCW) for each participant, thereby accounting for the informative censoring triggered by cloning. A Cox regression model was then performed to best account for potential biases and to obtain an estimate of the effect of DT over TT on VF (stratified for the presence of RAMs, as previously explained).

For a sensitivity analysis, we repeated the same procedure considering different grace periods—1, 2, and 3 years of viral suppression—to test the hypothesis that allowing for a longer grace period (and thus a potentially longer time of virologic suppression) could dilute the effect of RAMs on VF during the DT regimen.

## RESULTS

A cohort of 626 people was eligible for study analysis: 204 (32.6%) switched to DT, whereas 422 (67.4%) switched to the dolutegravir-based TT. Although this study is based on a multicenter cohort, the strict inclusion criteria limited the enrollment of all patients starting these strategies, especially DT. The reasons behind the switch to dolutegravir were different according to the treatment arm (*P* < .001) and were mainly represented by a proactive switch (for those switching to TT, 37.4%; for those switching to DT, 36.8%), toxicity (TT, 19.4%; DT, 29.9%), drug interactions (TT, 2.4%; DT, 6.4%).

The cohort was mainly composed of men (73%), with a median age of 45 years at baseline (ie, first HIV RNA <50 copies/mL), with no significant differences between the study groups. However, differences between the DT and TT groups were evidenced at baseline (ie, at the moment of the first HIV RNA <50 copies/mL during a protease inhibitor– or NNRTI-based 3-drug regimen), particularly concerning HIV duration and cumulative exposure to antiretrovirals (both longer in the TT group), nadir CD4 count (lower in the TT group), prevalence of RAMs (greater with TT), and HIV-1 viral subtype (non–B subtypes more frequent in DT group). Table 1 summarizes differences between the groups.

**Table 1.** Characteristics of Study Population at Baseline

	Arm, Median (IQR) or No. (%)		
Variable	Triple Therapy (n = 422)	Dual Therapy (n = 204)	*P* Value^a^
Age, y	45 (44–46)	44 (42–45)	.299
Male sex	297 (70.4)	160 (78.4)	.**033**
Risk factor for HIV infection			**<**.**001**
** **Heterosexual	176 (41.7)	88 (43.1)	
** **Men who have sex with men	140 (33.2)	90 (44.1)	
** **People who inject drugs	64 (15.1)	23 (11.3)	
** **Other/unknown	42 (10.0)	3 (1.5)	
Caucasian ethnicity	333 (78.9)	159 (77.9)	.965
Years since HIV diagnosis	5 (1–13)	3 (1–9)	.**002**
Years of antiretroviral exposure	2 (1–10)	1 (0–5)	**<**.**001**
Zenith HIV RNA, log_10_ copies/mL	4.57 (4.44–4.70)	4.73 (4.58–4.88)	.136
Nadir CD4 count, cells/μL	217 (200–234)	267 (246–289)	.**001**
Previous virologic failure, at least 1	47 (11.1)	22 (10.8)	.895
CDC stage C	53 (12.6)	14 (6.7)	.**031**
Anti-HCV–positive serostatus	74 (17.5)	21 (10.3)	.**018**
RAMs to study drugs at historical genotype, at least 1	87 (20.6)	18 (8.8)	**<**.**001**
B viral subtype	346 (82.0)	156 (76.5)	.**010**
Antiretroviral therapy			**<**.**001**
** **2 NRTIs + NNRTI	155 (36.7)	119 (58.3)	
** **2 NRTIs + bPI	267 (63.3)	85 (41.7)	
Backbone used in the switch regimen:		…	…
** **TFV/FTC	118 (28.0)		
** **ABC/3TC	304 (72.0)		

First HIV RNA <50 copies/mL before switch to 2 NRTIs + dolutegravir or 3TC/dolutegravir.

Abbreviations: 3TC, lamivudine; ABC, abacavir; bPI, boosted protease inhibitor; CDC, Centers for Disease Control and Prevention; FTC, emtricitabine; HCV, hepatitis C virus; NNRTI, nonnucleoside reverse transcriptase inhibitor; NRTI, nucleoside reverse transcriptase inhibitor; RAM, resistance-associated mutation; TFV, tenofovir.

^a^Bold indicates *P* < .05.

Concerning RAMs, 18 patients (8.8%) switching to DT harbored a virus with at least 1 RAM to NRTIs: 9 patients with only TAMs, 7 with M184V/I without TAMs, 1 with TAMs and M184V/I, and 1 with non-TAMs other than M184V/I (K65R/E/N). In the TT group, 87 (20.6%) patients harbored a virus with at least 1 RAM to NRTIs: 31 with only TAMs, 18 with M184V/I without TAMs, 34 with TAMs and M184V/I, and 4 with non-TAMs other than M184V/I. More detailed virologic characteristics of patients harboring a mutated virus are presented in Supplementary Table 1.

Patients switching to DT had a median 4.4 years of viral suppression, whereas those switching to a dolutegravir-based TT had a median 3.6 years of viral suppression (*P* < .001). Particularly, 8 patients (3.9%) switched to DT within 6 months of viral suppression and were followed up for a median 1.3 years; 13 (6.4%) switched within the first year and were followed up for a median 1.7 years; and 42 (24.9%) and 65 (31.9%) switched within the second and third years, with median follow-up times of 2.3 and 2.4 years, respectively. Patients switching to a dolutegravir-based TT were followed up for a median 1.4 years after the switch.

**Figure 1.**
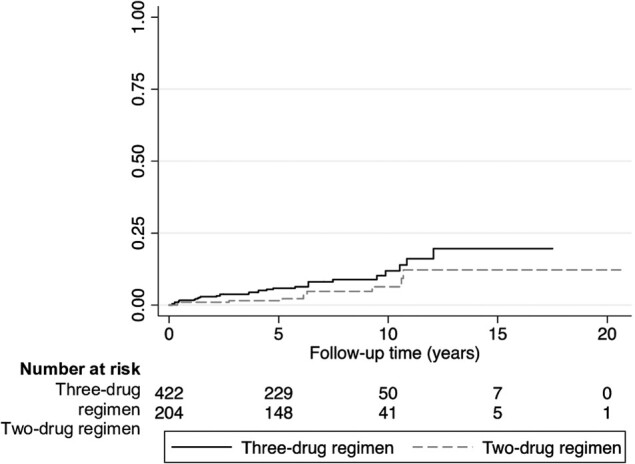
Kaplan-Meier estimate of time to virologic failure according to treatment arm (log-rank *P* = .073).

Overall 41 VFs (10 with DT, 31 with TT) occurred over a median follow-up of 5.8 years. Kaplan-Meier estimates showed a reduced risk of VF for the DT that was borderline significant (log-rank *P* = .073), as shown in Figure 1. In fact, risk of VF in the TT group was 1.7% at 6 months and 1 year, 2.9% at 2 years, and 3.7% at 3 years, whereas for the DT group, the risk of VF was 1.0% at 6 months and 1 and 2 years and 1.5% at 3 years. However, when we analyzed the risk of VF through the IPCW Cox model (selecting 6 months as the grace period for switching to a DT), the risk of VF was similar between DT and TT if no RAMs to the backbone were present at historical genotype (DT vs TT; adjusted hazard ratio [aHR], 0.88; 95% CI, .29–2.61; *P* = .812). Also, in a context of previously detected TAMs without M184V/I (DT vs TT; aHR, 3.41; 95% CI, .50–23.43; *P* = .211) and previously detected M184V/I without TAMs (DT vs TT; aHR, 3.06; 95% CI, .45–20.84; *P* = .252), DT performed similarly to TT, even if the point estimate of the hazard ratio suggested a trend for a higher risk of VF in the DT arm. Tables 2 and 3 summarize the results of the Cox regression analysis.

**Table 2.** IPCW-Adjusted Cox Regression Exploring the Role of RAMs to the Backbone on the Hazard of Virologic Failure: At Least 1 RAM vs None

	Model A		Model B	
Variable	aHR (95% CI)	*P* Value^a^	aHR (95% CI)	*P* Value
TT				
** **No mutations	1 [Reference]	…	0.74 (.35–1.56)	.428
** **At least 1 RAM	1.35 (.64–2.85)	.428	1 [Reference]	…
DT				
** **No mutations	0.88 (.29–2.61)	.812	0.65 (.17–2.53)	.533
** **At least 1 RAM	3.55 (1.08–11.68)	**.037**	2.63 (.62–11.16)	.190

With TT and no RAMs being the reference category in model A and TT with RAMs the reference category in model B.

Abbreviations: aHR, adjusted hazard ratio; DT, dual therapy; IPCW, inverse probability of censoring weight; RAM, resistance-associated mutation; TT, triple therapy.

^a^Bold indicates *P* < .05.

**Table 3.** IPCW-Adjusted Cox Regression Exploring the Role of RAMs to the Backbone on the Hazard of Virologic Failure: TAMs, M184V/I, TAMs + M184V/I, and Other Mutations to NRTIs

	Model A		Model B	
Variable	aHR (95% CI)	*P* Value^a^	aHR (95% CI)	*P* Value
TT				
** **No RAMs	1 [Reference]	…	0.41 (.14–1.18)	.099
** **With TAMs, no M184V/I	1.40 (.49–4.02)	.533	0.57 (.14–2.27)	.425
** **With M184V/I, no TAMs	2.46 (.84–7.15)	.099	1 [Reference]	…
** **With TAMs and M184V/I	NA	…	NA	…
** **With other RAMs	6.30 (1.46–27.09)	**.013**	2.56 (.47–14.08)	.279
DT				
** **No RAMs	0.89 (.30–2.64)	.826	0.36 (.07–1.76)	.207
** **With TAMs, no M184V/I	4.78 (1.03–22.14)	**.046**	1.94 (.29–13.24)	.497
** **With M184V/I, no TAMs	7.53 (1.63–34.73)	**.010**	3.06 (.45–20.84)	.252
** **With TAMs and M184V/I	NA	…	NA	…
** **With other RAMs	NA	…	NA	…

In model A, the reference category is TT with no RAMs; in model B, the reference category is TT + M184V/I and no TAMs.

Abbreviations: aHR, adjusted hazard ratio; DT, dual therapy; IPCW, inverse probability of censoring weight; NA, not applicable; NRTI, nucleoside reverse transcriptase inhibitor; RAM, resistance-associated mutation; TAM, thymidine analogue mutation; TT, triple therapy.

^a^Bold indicates *P* < .05.

Results from the IPCW Cox regression after choosing different grace periods yielded overall similar trends of associations at 1- and 2-year grace period analysis. By choosing 1 year as the grace period, the effect size of the associations was nearly identical to the 6-month period. In the 2-year grace period analysis, the trend for an increased risk of VF was confirmed with the use of DT in the setting of archived M184V/I and no TAMs (vs TT and M184V/I with no TAMs; aHR, 5.33; 95% CI, .78–36.46; *P* = .088) but was markedly reduced in the 3-year grace period analysis (DT vs TT and M184V/I with no TAMs; aHR, 2.33; 95% CI, .45–12.23; *P* = .317).

## DISCUSSION

The present study compared the efficacy of switching to lamivudine plus dolutegravir vs maintaining a 3-drug regimen after reaching virologic suppression. In line with randomized trials [4, 5], the DT regimen was highly effective in a less selected cohort from clinical practice. Particularly, no statistically significant difference could be found in the setting of archived RAMs, even if the low number of VFs prevented us from drawing definite conclusions.

This study is not the first to investigate the role of RAMs on the efficacy of the 2-drug regimens. A recent meta-analysis [6] on the efficacy of lamivudine plus dolutegravir, including evidence from randomized trials and real-world studies in a context of archived M184V/I mutations, reported a low incidence of VF at 2 years (<4%). Yet, 2 recent cohort studies raised the concern about the impact of RAMs on the potency of dual regimens with dolutegravir. Gagliardini et al [13] showed a similar effect of DT (including lamivudine and rilpivirine as companion drugs for dolutegravir) and TT on the incidence of VF, but an effect modification by low-level resistance to NRTIs was also evidenced, making the risk of failing 3 times higher for DT as compared with TT. Another study on ARCA [14] provided evidence of an increased risk of failure for patients taking lamivudine plus dolutegravir when RAMs were present in the backbone of DT and TT, particularly in the setting of M184V/I; however, the low incidence of VF resulted in a very low precision of the estimates.

Other studies did not confirm those associations between cumulative genotypic resistance tests and VF during lamivudine plus dolutegravir. Particularly, in the small pilot ART-PRO trial [8] on patients switching to lamivudine plus dolutegravir after virologic suppression, the presence of historical resistance to lamivudine did not have any impact on the rates of VF during 144 weeks. However, the limited sample size (41 participants) hampered the authors to draw definite conclusions. More recently, the SOLAR-3D [9] prospective trial evaluated lamivudine plus dolutegravir as a simplification strategy in 100 patients with previous VFs. The authors confirmed a high proportion of virologic suppression independently from the presence of M184V/I at historical genotype. Yet, it should be noted that the time of virologic suppression was strikingly longer for patients with previous M184V/I detected at a genotypic resistance test (12.8 vs 8.9 years). This is an important factor when analyzing observational data. In fact, patients starting a DT usually have more favorable characteristics than those starting a TT in the setting of regimen optimization. Some of these characteristics (eg, nadir CD4 count or pretreatment HIV RNA) are known confounders for which regression models can be adjusted. Other important variables, such as time of persistent virologic suppression, cannot be accounted by adjustment, since they represent a real selection bias. The major strength of our study is therefore the attempt to correct for this bias, as illustrated in previous articles [15, 18]. Other important strengths include the large sample size of the study population, representing different clinical centers in Italy, and the adjustment for multiple factors in our analysis, rendering the 2 treatment groups more comparable in baseline characteristics.

Some limitations have to be acknowledged. First, the low number of patients switching to DT (especially within the first 6 months) and the low incidence of VF reduced the precision of the estimates concerning the impact of the exposure variables. This was not unexpected, due to the strict inclusion and exclusion criteria (among which the requirement of at least 1 genotypic resistance test before baseline). Yet, the selection of the cohort allowed comparison of DT and TT in some important characteristics, such as RAMs, that is not always possible in observational studies. Another study limitation is the possibility of a number of unknown confounders not taken into account in the analysis: despite adjustment for measurable variables (ie, sociodemographic characteristics, cumulative duration of antiviral exposure, nadir CD4 count, zenith HIV RNA, HIV viral subtype, number of previous VFs, RAMs, hepatitis C virus serostatus, previous AIDS-defining event), important covariates, such as adherence to antiretroviral therapy, could not be explored and accounted in the analysis. Last, the choice of a stringent definition for VF could be questioned due its potential to confuse viral blips or low-level viremia with true VFs. However, recent data suggest that low-level viremia and, at a lesser extent, viral blips could represent a risk factor for subsequent VF [19]; also, in a multicohort collaboration [20], resistance mutations to dolutegravir appeared more frequently at failure of lamivudine plus dolutegravir than after failure of a dolutegravir-based 3-drug regimen. Together, these considerations prompted us to consider a highly sensitive outcome that could overcome the low incidence of real VFs expected with the 2 strategies.

Despite the limitations, some important conclusions can be drawn from our observations. The results of our study showed that DT is not inferior to TT. This result strongly supports the use of the DT in a real-life setting for a broad range of patients. However, despite the lack of precision due to large confidence intervals of the estimates, an association between DT and a higher risk of VF could not be completely ruled out when RAMs are combined with a shorter time (≤2 years) of continuous viral suppression: indeed, a larger sample size, with a calculated number of 26 VFs, would have led the estimated aHR for VF to be statistically significant at an α error of 0.05. Conversely, allowing for a 3-year grace period before switching to DT seemed to nullify the borderline statistically significant effect of archived RAMs on the efficacy of this simplification strategy. Therefore, pending longer follow-up studies with larger population, a cautious approach toward simplifying treatment is advisable: particularly, the choice of switching to a DT in a patient with previous RAMs could be considered only when other individual characteristics have been consistent over time, including treatment adherence and compliance with follow-up visits.

## Supplementary Material

ofae198_Supplementary_Data

## Supplementary Data

Supplementary materials are available at *Open Forum Infectious Diseases* online. Consisting of data provided by the authors to benefit the reader, the posted materials are not copyedited and are the sole responsibility of the authors, so questions or comments should be addressed to the corresponding author.

## Notes

***Acknowledgments*.** S. D. G. contributed to study concept and data interpretation; A. B. contributed to study concept, data analysis and interpretation and drafted the article; A. C., F. Lombardi, R. A. P., F. Lamanna, D. F., A. D., and G. B. contributed to data collection and revised the article; D. G. and A. C. substantially contributed to data analysis; M. Z. contributed to data interpretation. All authors approved the final version.

***Data-sharing statement.*** All data and commands used to perform study analysis on Stata can be made available upon reasonable request.

***Patient consent statement.*** The study was performed in accordance with the 1964 Declaration of Helsinki and later amendments. All patients signed an informed consent for use of their clinical and laboratory data in aggregated and anonymous form. The study protocol was authorized by the ethics committee of the promoter center (South-East Tuscany Ethics Committee, protocol code ARCA, 21 July 2014) and then approved by the local institutional ethics committees of the participating centers in agreement with Italian and European privacy legislation.

***Financial support.*** This study was performed as part of our routine activity. No specific funding was used.
