# Supplementary material for: Efficacy of Lamivudine Plus Dolutegravir vs Dolutegravir-Based 3-Drug Regimens in People With HIV Who Are Virologically Suppressed
Source: Open Forum Infect Dis. 2024 Apr 10;11(5):ofae198. doi: 10.1093/ofid/ofae198 (PMC11055392; doi:10.1093/ofid/ofae198)
Supplement: ofae198_Supplementary_Data [file ofae198_supplementary_data.docx]

**Supplementary table 1.** Characteristics of patients harboring a virus with previously detected resistance-associated mutations.

| **Patient** | **Switch therapy** | **Resistance-associated mutations** | **GSS score 3TC (FTC)** | **GSS score ABC/TFV** | **Interpretation of resistance** | **Time of virological suppression before switch** | **Virological failure** |
| --- | --- | --- | --- | --- | --- | --- | --- |
| 1 | 3TC+DTG | D67N, K219Q/E, K70R, M41L, T215F/Y | 15 |  | Low level | 8.4 | 0 |
| 2 | 3TC+DTG | K65R/E/N | 30 |  | Intermediate | 9.9 | 0 |
| 3 | 3TC+DTG | D67N, F116Y, K219Q/E, K70R | 15 |  | Low-level | 9.2 | 1 |
| 4 | 3TC+DTG | T215F/Y | 0 |  | Susceptible | 0.7 | 0 |
| 5 | 3TC+DTG | M184V | 60 |  | High level | 1.1 | 0 |
| 6 | 3TC+DTG | A62V, M184V | 60 |  | High level | 2.5 | 0 |
| 7 | 3TC+DTG | M41L | 0 |  | Susceptible | 1.2 | 0 |
| 8 | 3TC+DTG | D67N, K70R | 0 |  | Susceptible | 3.6 | 0 |
| 9 | 3TC+DTG | M184V | 60 |  | High level | 2.8 | 0 |
| 10 | 3TC+DTG | M184V | 60 |  | High level | 14.7 | 0 |
| 11 | 3TC+DTG | M184V | 60 |  | High level | 1.1 | 1 |
| 12 | 3TC+DTG | A62V, M184V | 60 |  | High level | 10.8 | 0 |
| 13 | 3TC+DTG | T215F/Y, M184V, M41L | 65 |  | High level | 19.3 | 0 |
| 14 | 3TC+DTG | D67N, K219Q/E, K70R | 10 |  | Potential low-level | 11.4 | 0 |
| 15 | 3TC+DTG | T215F/Y, M41L | 5 |  | Susceptible | 5.4 | 0 |
| 16 | 3TC+DTG | M41L | 0 |  | Susceptible | 1.2 | 0 |
| 17 | 3TC+DTG | D67N, K70R | 0 |  | Susceptible | 3.6 | 0 |
| 18 | 3TC+DTG | M184V | 60 |  | High level | 14.4 | 0 |
| 1 | TFV/FTC/DTG | T215F/Y, D67N, M184V, M41L | 65 | 25 | 3TC: high level  TDF: low level | 1.4 | 0 |
| 2 | ABC/3TC/DTG | A62V, M184V | 60 | 20 | 3TC: high level  ABC: low level | 6.1 | 0 |
| 3 | ABC/3TC/DTG | V75I | 5 | 5 | Susceptible | 5.4 | 0 |
| 4 | ABC/3TC/DTG | M184V | 60 | 15 | 3TC: high level  ABC: low level | 5.8 | 0 |
| 5 | TFV/FTC/DTG | L210W, M41L | 0 | 20 | 3TC: susceptible  TDF: low level | 8.9 | 0 |
| 6 | TFV/FTC/DTG | K219Q/E, K70R | 0 | 10 | 3TC: susceptible  TDF: potential low level | 3.4 | 0 |
| 7 | TFV/FTC/DTG | T215F/Y | 0 | 10 | 3TC: susceptible  TDF: potential low-level | 1.4 | 0 |
| 8 | ABC/3TC/DTG | K219Q/E, K70R, M41L | 0 | 15 | 3TC: susceptible  ABC: low-level | 1.3 | 0 |
| 9 | ABC/3TC/DTG | K65R/E/N | 40 | 45 | 3TC: intermediate  ABC: intermediate | 1.4 | 0 |
| 10 | ABC/3TC/DTG | T215F/Y, D67N, K219Q/E, K70R | 10 | 40 | 3TC: potential low  ABC: intermediate | 0.9 | 0 |
| 11 | ABC/3TC/DTG | T215F/Y, M184V, M41L | 65 | 50 | 3TC: high level  ABC: intermediate | 3.8 | 0 |
| 12 | TFV/FTC/DTG | T215F/Y, D67N, K219Q/E, K70R, M184V, M41L | 75 | 50 | 3TC: high level  TDF: intermediate | 3.5 | 0 |
| 13 | TFV/FTC/DTG | V75I, F77L | 10 | 10 | 3TC: potential low  TDV: potential low | 0.5 | 1 |
| 14 | TFV/FTC/DTG | T215F/Y, M184V, M41L | 65 | 15 | 3TC: high level  TFV: low level | 5.2 | 0 |
| 15 | TFV/FTC/DTG | T215F/Y, V75I, D67N, F77L, K219Q/E, K70R, L210W, M184V, M41L | 100 | 95 | 3TC: high level  TFV: high level | 6.4 | 0 |
| 16 | ABC/3TC/DTG | D67N, K219Q/E, K70R | 10 | 25 | 3TC: potential low  ABC: low level | 7.4 | 0 |
| 17 | TFV/FTC/DTG | T215F/Y, L210W, M184V, M41L | 80 | 50 | 3TC: high level  TDF: intermediate | 7.5 | 0 |
| 18 | TFV/FTC/DTG | M184V | 60 | -10 | 3TC: high level  TFV: susceptible | 5.3 | 1 |
| 19 | ABC/3TC/DTG | K70R | 0 | 5 | susceptible | 11.2 | 0 |
| 20 | TFV/FTC/DTG | T215F/Y, L210W, M41L | 20 | 60 | 3TC: low level  TFV: high level | 4.9 | 0 |
| 21 | ABC/3TC/DTG | T215F/Y, M184V, M41L | 65 | 50 | 3TC: high level  ABC: intermediate | 9.0 | 0 |
| 22 | TFV/FTC/DTG | K219Q/E, K70R, M184V | 60 | 0 | 3TC: high level  TFV: susceptible | 8.9 | 0 |
| 23 | TFV/FTC/DTG | T215F/Y, M184V, M41L | 65 | 15 | 3TC: high level  TFV: low level | 2.4 | 0 |
| 24 | TFV/FTC/DTG | T215F/Y, D67N, K219Q/E, K70R, M184V, M41L | 75 | 50 | 3TC: high level  TFV: intermediate | 9.7 | 0 |
| 25 | ABC/3TC/DTG | K70E | 10 | 15 | 3TC: potential low level  ABC: low level | 3.5 | 1 |
| 26 | TFV/FTC/DTG | A62V, M184V | 60 | -5 | 3TC: high level  TFV: susceptible | 0.9 | 0 |
| 27 | TFV/FTC/DTG | M41L | 0 | 5 | Susceptible | 7.1 | 0 |
| 28 | TFV/FTC/DTG | M184V | 60 | -10 | 3TC: high level  TFV: susceptible | 2.8 | 0 |
| 29 | ABC/3TC/DTG | K70R | 0 | 5 | susceptible | 8.9 | 0 |
| 30 | TFV/FTC/DTG | A62V, K219Q/E, K70R, M184V | 60 | 5 | 3TC: high level  TFV: susceptible | 4.3 | 0 |
| 31 | ABC/3TC/DTG | T215F/Y, L210W, M41L | 20 | 60 | 3TC: low level  ABC: high level | 4.5 | 0 |
| 32 | ABC/3TC/DTG | K219Q/E, M184V | 60 | 20 | 3TC: high level  ABC: low level | 7.3 | 0 |
| 33 | ABC/3TC/DTG | T215F/Y, D67N, K219Q/E, K70R, L210W, M41L | 30 | 95 | 3TC: intermediate  ABC: high level | 7.9 | 0 |
| 34 | TFV/FTC/DTG | T215F/Y, D67N, L210W, M184V, M41L | 80 | 60 | 3TC: high level  TFV: high level | 4.3 | 0 |
| 35 | ABC/3TC/DTG | M184V, M41L | 60 | 20 | 3TC: high level  ABC: low level | 6.5 | 0 |
| 36 | ABC/3TC/DTG | D67N, K219Q/E, K70R | 10 | 25 | 3TC: potential low level  ABC: low level | 5.0 | 0 |
| 37 | ABC/3TC/DTG | A62V, K65R/E/N, M184V | 90 | 65 | 3TC: high level  ABC: high level | 5.2 | 0 |
| 38 | TFV/FTC/DTG | T215F/Y, M184V, M41L | 65 | 15 | 3TC: high level  TFV: low level | 6.0 | 0 |
| 39 | ABC/3TC/DTG | M184V | 60 | 15 | 3TC: high level  ABC: low level | 3.6 | 0 |
| 40 | TFV/FTC/DTG | T215F/Y, L210W, M184V, M41L | 80 | 50 | 3TC: high level  TFV: intermediate | 1.4 | 0 |
| 41 | TFV/FTC/DTG | T215F/Y, K65R/E/N, L210W, M184V | 90 | 65 | 3TC: high level  TFV: high level | 4.3 | 0 |
| 42 | ABC/3TC/DTG | T215F/Y, L210W, M184V, M41L | 80 | 85 | 3TC: high level  ABC: high level | 2.9 | 0 |
| 43 | ABC/3TC/DTG | Q151M, F77L, M184V | 80 | 90 | 3TC: high level  ABC: high level | 3.1 | 0 |
| 44 | TFV/FTC/DTG | K219Q/E | 0 | 5 | susceptible | 0.0 | 1 |
| 45 | ABC/3TC/DTG | K70R, M184V | 60 | 20 | 3TC: high level  ABC: low level | 5.1 | 0 |
| 46 | ABC/3TC/DTG | D67N, K219Q/E, K70R | 10 | 25 | 3TC: potential low level  ABC: low level | 2.3 | 0 |
| 47 | ABC/3TC/DTG | A62V, M184V | 60 | 20 | 3TC: high level  ABC: low level | 3.6 | 0 |
| 48 | ABC/3TC/DTG | T215F/Y, L210W, M41L | 20 | 60 | 3TC: low level  ABC: high level | 4.1 | 0 |
| 49 | ABC/3TC/DTG | T215F/Y, M184V | 60 | 25 | 3TC: high level  ABC: low level | 0.3 | 0 |
| 50 | ABC/3TC/DTG | K70R, M184V | 60 | 20 | 3TC: high level  ABC: low level | 3.7 | 0 |
| 51 | ABC/3TC/DTG | A62V, M184V | 60 | 20 | 3TC: high level  ABC: low level | 0.0 | 1 |
| 52 | ABC/3TC/DTG | D67N, K219Q/E, K70R, M184V | 70 | 50 | 3TC: high level  ABC: intermediate | 3.9 | 0 |
| 53 | ABC/3TC/DTG | D67N, K219Q/E, K70R | 10 | 25 | 3TC: potential low level  ABC: low level | 1.0 | 0 |
| 54 | TFV/FTC/DTG | M41L | 0 | 5 | Susceptible | 2.4 | 0 |
| 55 | ABC/3TC/DTG | M184V | 60 | 15 | 3TC: high level  ABC: low level | 0.7 | 0 |
| 56 | ABC/3TC/DTG | T215F/Y, D67N, L210W, M41L | 20 | 70 | 3TC: low level  ABC: high level | 3.7 | 0 |
| 57 | TFV/FTC/DTG | T215F/Y, D67N, L210W, M184V, M41L | 80 | 60 | 3TC: high level  TFV: low level | 2.6 | 0 |
| 58 | ABC/3TC/DTG | D67N, K219Q/E | 0 | 10 | 3TC: susceptible  ABC: potential low level | 1.7 | 0 |
| 59 | ABC/3TC/DTG | T215F/Y, D67N, K219Q/E, K70R, M41L | 15 | 60 | 3TC: low level  ABC: high level | 8.3 | 0 |
| 60 | ABC/3TC/DTG | M41L | 0 | 5 | susceptible | 5.6 | 0 |
| 61 | ABC/3TC/DTG | T215F/Y, K219Q/E, K70R, M184V, M41L | 65 | 60 | 3TC: high level  ABC: high level | 10.5 | 0 |
| 62 | TFV/FTC/DTG | T215F/Y, L210W, M184V, M41L | 80 | 50 | 3TC: high level  TFV: intermediate | 10.1 | 0 |
| 63 | ABC/3TC/DTG | T215F/Y, K70R, L210W | 0 | 30 | 3TC: susceptible  ABC: intermediate | 14.7 | 0 |
| 64 | ABC/3TC/DTG | T215F/Y, M41L | 5 | 25 | 3TC: susceptible  ABC: low level | 10.2 | 0 |
| 65 | TFV/FTC/DTG | T215F/Y, K65R/E/N, L210W, M184V | 90 | 65 | 3TC: high level  TFV: high level | 5.7 | 0 |
| 66 | ABC/3TC/DTG | T215F/Y, K70R, L210W | 0 | 30 | 3TC: susceptible  ABC: intermediate | 8.1 | 0 |
| 67 | ABC/3TC/DTG | T215F/Y, L210W, M184V, M41L | 80 | 85 | 3TC: high level  ABC: high level | 8.8 | 0 |
| 68 | ABC/3TC/DTG | Q151M, F77L, M184V | 80 | 90 | 3TC: high level  ABC: high level | 9.0 | 0 |
| 69 | ABC/3TC/DTG | M184V | 60 | 15 | 3TC: high level  ABC: low level | 1.8 | 0 |
| 70 | ABC/3TC/DTG | M184V | 60 | 15 | 3TC: high level  ABC: low level | 6.7 | 0 |
| 71 | TFV/FTC/DTG | K219Q/E | 0 | 5 | susceptible | 0.0 | 1 |
| 72 | ABC/3TC/DTG | K70R, M184V | 60 | 20 | 3TC: high level  ABC: low level | 12.6 | 0 |
| 73 | ABC/3TC/DTG | D67N, K219Q/E, K70R | 10 | 25 | 3TC: potential low level  ABC: low level | 13.5 | 0 |
| 74 | ABC/3TC/DTG | A62V, M184V | 60 | 20 | 3TC: high level  ABC: low level | 4.8 | 0 |
| 75 | ABC/3TC/DTG | T215F/Y, L210W, M41L | 20 | 60 | 3TC: low level  ABC: high level | 16.9 | 0 |
| 76 | ABC/3TC/DTG | T215F/Y, M184V | 60 | 25 | 3TC: high level  ABC: low level | 0.3 | 0 |
| 77 | ABC/3TC/DTG | K70R, M184V | 60 | 20 | 3TC: high level  ABC: low level | 6.5 | 0 |
| 78 | ABC/3TC/DTG | A62V, M184V | 60 | 20 | 3TC: high level  ABC: low level | 0.0 | 1 |
| 79 | ABC/3TC/DTG | K70R, M184V | 60 | 20 | 3TC: high level  ABC: low level | 7.2 | 0 |
| 80 | ABC/3TC/DTG | T215F/Y, K65R/E/N, M184V, M41L | 95 | 95 | 3TC: high level  ABC: high level | 11.4 | 0 |
| 81 | ABC/3TC/DTG | D67N, K219Q/E, K70R, M184V | 70 | 50 | 3TC: high level  ABC: intermediate | 7.8 | 0 |
| 82 | ABC/3TC/DTG | D67N, K219Q/E, K70R | 10 | 25 | 3TC: potential low level  ABC: low level | 1.0 | 1 |
| 83 | TFV/FTC/DTG | M41L | 0 | 5 | Susceptible | 5.8 | 0 |
| 84 | ABC/3TC/DTG | M184V | 60 | 15 | 3TC: high level  ABC: low level | 14.6 | 0 |
| 85 | ABC/3TC/DTG | M184V | 60 | 15 | 3TC: high level  ABC: low level | 0.7 | 0 |
| 86 | ABC/3TC/DTG | T215F/Y, D67N, L210W, M41L | 20 | 70 | 3TC: low level  ABC: high level | 12.6 | 0 |
| 87 | TFV/FTC/DTG | T215F/Y, D67N, L210W, M184V, M41L | 80 | 60 | 3TC: high level  TFV: high level | 8.9 | 0 |
